# Selective inhibition of miR-21 by phage display screened peptide

**DOI:** 10.1093/nar/gkv185

**Published:** 2015-03-30

**Authors:** Debojit Bose, Smita Nahar, Manish Kumar Rai, Arjun Ray, Kausik Chakraborty, Souvik Maiti

**Affiliations:** 1 Proteomics and Structural Biology Unit, Institute of Genomics and Integrative Biology, CSIR. Mathura Road, Delhi 110020, India; 2 Academy of Scientific and Innovative Research (AcSIR), Anusandhan Bhawan, 2 Rafi Marg, New Delhi-110001, India; 3 National Chemical Laboratory, CSIR, Dr. Homi Bhabha Road, Pune 411008, India

## Abstract

miRNAs are nodal regulators of gene expression and deregulation of miRNAs is causally associated with different diseases, including cancer. Modulation of miRNA expression is thus of therapeutic importance. Small molecules are currently being explored for their potential to downregulate miRNAs. Peptides have shown to have better potency and selectivity toward their targets but their potential in targeting and modulating miRNAs remain unexplored. Herein, using phage display we found a very selective peptide against pre-miR-21. Interestingly, the peptide has the potential to downregulate miR-21, by binding to pre-miR-21 and hindering Dicer processing. It is selective towards miR-21 inside the cell. By antagonising miR-21 function, the peptide is able to increase the expression of its target proteins and thereby increase apoptosis and suppress cell proliferation, invasion and migration. This peptide can further be explored for its anti-cancer activity in vivo and may be even extended to clinical studies.

## INTRODUCTION

MicroRNAs are small noncoding RNAs, playing crucial roles in regulating cellular function via post transcriptional gene regulation (1). They are transcribed to form a long precursor miRNA(pri-miRNA) which gets processed inside nucleus by microprocessor complex to form stem loop intermediate of around 70 nt long (pre-miRNA) which then get exported to cytoplasm via exportin and again gets processed to form a mature miRNA duplex. Mostly, the strand with relatively less thermodynamic stability in the duplex, the guide strand (mature miRNA) gets incorporated to RISC complex and binds to 3′ UTR of its target and causes translational suppression and/or mRNA decay (2).

miRNAs control as much as 60% of all human genes (3) and play a pivotal role in different cellular processes, including development, stress response, cell proliferation, apoptosis, immunity, hematopoiesis and induction and maintenance of pluripotency (4–7). Dysregulated miRNA expression has been causally associated with different disease pathologies, including cancer, diabetes, cardiovascular disease, neurological disorder etc (8–10). In case of cancer, around 40% of miRNAs are overexpressed (11,12) and are shown to be involved in cancer initiation, progression, metastasis and resistance to therapeutic drugs (13). Thus, modulation of miRNA expression is of therapeutic importance. The well-known miRNA inhibitors are oligonucleotide based antisense oligonucleotide (anti-miR), sponges and DNAzyme (14–16). Though they are ideal tools for deciphering the role of a specific miRNA in disease context, their suboptimal delivery, low scalability, unfavorable pharmacodynamic and pharmacokinetic properties are some of the major hurdles in their therapeutic application (17). This paves the way for exploring alternative strategies for miRNA modulation. Very recently, different groups, including us, have shown promising impact of small molecules in modulating miRNA expression and its function (18–31). Though this small molecule mediated modulation of miRNA is a potentially important area to explore, adequate selectivity is difficult to attain. Small molecule inhibitors against a particular miRNA have been termed as selective by checking its effect only on a very small subset of miRNAs. MiRnome-wide effect of these miRNA inhibitors have to be explored and parallel efforts have to be made to look for a class of miRNA inhibitors that are intrinsically far more selective. Last decade has seen enormous growth in peptide based drugs because of their exquisite potency and selectivity toward their molecular targets. Peptides are very well reported to selectively bind to RNA (32). This gave us a clue to look for selective peptide inhibitors against miRNA. miR-21 is one of the most well studied miRNA, which is consistently upregulated in different types of cancer. Conditional over expression and inactivation of miR-21 was directly correlated with tumor formation and regression in mice model, showing its oncogenic potential (33). miR-21 is known to play major role in cancer progression by favoring cancer cell invasion, migration and proliferation, metastasis and also by preventing apoptosis (34–36). Thus, miR-21 is a strong candidate miRNA to target in case of cancer and downregulation of miR-21 and modulation of its function is of promising therapeutic importance.

Recently, phage display technology has allowed us to find selective peptide(s) against RNA of interest by screening a peptide library of around 10^9^ or more complexity (37,38). We have used phage display against pre-miR-21 to find out a selective peptide that binds to pre-miR-21 and block Dicer processing and thereby downregulate miR-21 expression. This peptide is very selective to miR-21 inside cell. We have also shown that the peptide inhibitor increases apoptosis, suppress cell invasion and migration.

## MATERIALS AND METHODS

### *In vitro* transcription of pre-miR-21

The pre-miR-21 RNA was *in vitro* transcribed as described previously (18). This was further gel purified using 15% denaturing polyacrylamide gel electrophoresis (PAGE).

### Biotinylation of pre-miR-21

The PAGE purified pre-miR**-**21 was biotinylated using Thermo Scientific Pierce RNA 3′ End Biotinylation Kit using manufactures protocol.

### Phage display selection against pre-miR-21

We have used Ph.D.-12 library for the selection where 12 mer random peptide was fused to the N-terminus of minor coat protein pIII of M13 phage. The library has around 10^9^ complexity. Phage display selection was carried out as described previously (37). The entire panning was done at room temperature. For the initial negative selections, 25 μl of magnetic Dynabeads (M-280 streptavidin beads) were washed three times with 100 μl Solution A (0.1 M NaOH, 0.05 M NaCl), once with 100 μl of buffer B (0.1 M NaCl) and then once with RNA/phage washing buffer (10 mM Tris·HCl, pH 7.5, 10 mM MgCl2, 150 mM NaCl, 1 mM DTT). During each washing, the tubes were set on a magnetic holder while the supernatant was removed. 10 μl phage library (∼ 10^12^ pfu) was added to the beads, incubated for 30 min, and supernatant was removed. The bead bound phages were eluted as described in the protocol for the Ph.D.-12 library and titered. This negative selection was carried out to remove the streptavidin and tube binding phages. After four rounds of consecutive negative selections (supernatant was passed directly to the next round), we found no bead binders (there was no eluted phage while titrating for the bead binders, after fourth round of negative selection), suggesting that the final pool was devoid of any streptavidin or plate binder. Positive selection for binding to pre-miR-21 was carried out using this final depleted pool. Fifty pmoles (or 30 pmoles in third and fourth rounds) of biotinylated pre-miR-21 were added to pre-washed beads and incubated for 30 min at RT. Pre-miR-21 bound tubes were washed with RNA/phage binding buffer A, pre-screened phage library was added to it and incubated at room temperature for 30 min. Tubes were washed with 200 μl of RNA/phage washing buffer containing 0.1% Tween-20. Bound phages were eluted by glycine (0.2 M glycine, pH 2.2). The eluted phage pool was amplified in *Escherichia coli* ER2738 and the procedure was repeated for subsequent rounds, without additional pre-screening. The stringency of washing was increased, by increasing the number of washes and amount of Tween-20, in consecutive rounds. To increase the selectivity against pre-miR-21, the phages were pre-incubated with non-biotinylated pre-miR-27a and total RNA from HEK cell line (where miR-21 expression level is basal) in the third and fourth rounds of panning, respectively.

### Genomic DNA isolation and PCR amplification of insert

At the end of the fourth round, eluted phages were titered on IPTG/X-gal plate and genomic DNA was isolatedfrom randomly selected 20 colonies using DNA isolation protocol (Ph.D. Phage Display Libraries kit manual). The polymerase chain reaction (PCR) amplification was done to amplify 36 nt insert by using the forward primer- GCAAGCCTCAGCGACCGAAT and reverse primer- CCCTCATAGTTAGCGTAACG, which anneal ∼130 bp upstream and downstream of constant flanking region of insert (total amplicon length 360 bp) by using program in thermal cycler [95°C initial denaturation for 5 min, 35 cycles for 95°C denaturation 30 s, 55°C annealing for 30 s. and 72°C amplification 30 s and final extension at 72°C for 5 min using the fusion DNA polymerase enzyme]. The amplified PCR product was run on 1.5% agarose gel and band of our interest (360 bp) was gel purified by QIAquick Gel Extraction Kit. The final DNA amplicon was quantified using nanodrop and 500ng of each sample was sent for sequencing (RSCC Company). From sequencing result, we identified the sequence of interest which codes for the 12 amino acid residues peptide and translated into amino acid sequence using online available ExPASy: SIB Bioinformatics Resource Portal tool.

### Ion torrent sequencing

For ion torrent sequencing, the genomic DNA were isolated using Quiagen M13 genomic DNA isolation kit (QIAprep Spin M13 Kit) from amplified phage pool of fourth round selection. The fusion primers (adapter sequence which was used in sequencing and primer for amplification of insert) were used to amplify the 170 bp length of amplicon having 36 bp insert.

Forward primer (P1)-CCTCTCTATGGGCAGTCGGTGATTCGCAATTCCTTTAGTGGTACC,

reverse primer (A1_RP1)CCATCTCATCCCTGCGTGTCTCCGACTCAG ATCACGTTC TGTATGGGA TTTTGCTAAACAAC.

The fusion primers were designed in such a way that it contains primer sequence for amplification of insert as well as adapter sequence, which will help in sequencing. Details about the ion torrent library preparation and design of the fusion primer is available in ion torrent library preparation manual (Ion Amplicon Library Preparation (Fusion Method) Publication Number 4468326). The template was prepared from amplified PCR using ion one touch instrument and enrichment of positive ion sphere particle was done using Ion one touch ES instrument (Ion OneTouch™ Template Kit, 4468660). Details about the template preparation and enrichment of positive ion sphere particle is available in template preparation manual (Ion OneTouch™ Template Kit, Catalog Number 4468660). The prepared template was sequenced in ion torrent sequencer using the manual Ion Sequencing Kit User Guide v2.0, 4468997. We wrote a program in perl for analysis of the output fastQ file generated after sequencing, to find out the type of peptide enriched after fourth round of panning.

### Fluorescence spectroscopy

Intrinsic fluorescence spectra of the tryptophan containing peptides were measured in Fluoromax 4 (Spex) spectrofluorophotometer. The excitation wavelength was set at 285 nm, and the emission spectra were recorded from 330 to 400 nm. In the titration experiments, the peptide concentration was fixed at 100 nM in a buffer containing 10 mM Tris-HCl (pH 7.5), 10 mM NaCl, 1 mM MgCl_2_ and the concentrations of the pre-miRNAs were varied from 0 to 2 μM. The changes in the fluorescence intensity at 355 nm (maximum wavelength) were monitored as a function of pre-miRNA concentrations. For data analysis,

\begin{equation*} \Delta {\rm F} = {\rm F}/({\rm F}_{\rm i} - 1) \end{equation*}

where *F* is the observed fluorescence intensity at each titrant concentration and *F_i_* is the fluorescence intensities of initial states of titration. The data were fitted in the following equation:

\begin{equation*} {\rm y} = {\rm P}_1 {\rm x}({\rm P}_2 + {\rm x}) \end{equation*}

### Peptide modeling and Docking

Conformational modeling of the peptide was carried using PEP-FOLD (39). PEP-FOLD is based on concept of structural alphabets, which are assembled using greedy algorithm. Greedy algorithm is an approximation algorithm in which a global optimum solution is reached by making step-by-step local optimum choices. It is called ‘greedy’ due to its short-sighted approach of taking into account the best local optimal solutions in solving a problem rather than having a global overview. The models were ranked according to their GDT score. Global distance test (GDT) is a measure of model assessment by superimposing structural models of the same sequence and is calculated by the average score at difference distance cutoff. It is a widely accepted standard measure used in the Critical Assessment of protein Structure Prediction (CASP) experiments. To check for conformational stability, molecular dynamic simulation was done using GROMACS 4.6.1 (40). All atomistic simulations were carried out using the CHARMM27 all-atom force field (version 2.0) (41–43) using periodic boundary condition. The starting models were solvated in a periodic box with TIP3 water model. Na ions were added to the solvent to neutralize electrical net charge of the protein. Each system was then minimized for 50000 steps using a steepest decent algorithm. The NPT (the number of particles, system pressure and temperature conserved) ensemble was used for production simulation. Systems were simulated at 310K, maintained separately for proteins, lipids, water by a Berendsen thermostat with a time constant of 1 ps. Pressure coupling was done employing a Berendsen barostat using a 1 bar reference pressure and a time constant of 2 ps. Electrostatic interactions were calculated using the particle mesh Ewald (PME) summation.

The miRNA was modeled using mc-fold | mc-sym pipeline (44). Docking between the modeled peptide and miRNA was performed using Zdock 3.0.2 (45). ZDock uses a Fast Fourier Transformation (FFT) algorithm to search the rotational space. The models were ranked according to their ZDock score, which is based on a shape complimentary scoring function.

### Enzymatic footprinting

P^32-^5′ end labeled pre-miR-21 was subjected to a denaturation and renaturation procedure before structural probing. The P^32-^5′ end-labeled RNA (60000 cpm) was incubated in 10 mM Tris-HCl (pH 7.5), 10 mM NaCl, 1 mM MgCl_2_ in a total volume of 8 μl. The sample was heated to 90°C for 5 min and allowed for slow cooling to 37°C. 1 μl peptide was added to a final concentration of 200 nM, 500 nM and 1 μM and incubated at 37°C for 30 minutes. 1 μl of S1 nuclease (47.5 U) (Fermentas) was added and incubated at 37°C for 90 s. Reaction was stopped by adding 95% formamide with dyes followed by immediate snap chilling on dry ice. Alkaline digestion ladder and RNase T1 digestion ladder was generated as described by Regulski *et al*. (46). Samples were run in 15% denaturing PAGE and exposed to phosphor screen overnight. Images were obtained using Typhoon FLA 7000 phosphoimager. Cleavage profiles were generated using ImageQuant 5.2 software.

### Dicer blocking assay

P^32-^5′ end-labeled RNA (40000 cpm, ∼10 ng) was incubated in a buffer containing 10 mM sodium cacodylate, 1 mM MgCl_2_, and 10 mM NaCl (pH 7.5) in a total volume of 8 μl. The reaction mixture was heated at 90°C for 5 min, followed by slow cooling to 37°C. 1 μl peptide was added to a final concentration of 50 nM, 100 nM, 200 nM, 500 nM and 1 μM, incubated for 30 min followed by addition of 1 μl (0.05 units) of recombinant turbo Dicer (Genlantis). For mutant peptide, only 1 μM concentration was used. The assay was performed at 37°C for 1 h. Reaction was stopped by adding Dicer stop buffer (Genlantis). 10 μl 95% formamide with dyes (Xylene Cyanol and Bromophenol Blue) were added and ran in a 15% denaturing PAGE. Gel was exposed to phosphor screen overnight and image was taken using Typhoon FLA 7000 phosphoimager.

### Quantitative uptake of peptide inside the cell

Carboxyfluorescein (FAM)-conjugated peptide was added onto MCF-7 cells with ∼80% confluence in serum free media. Cells were then incubated at 37°C humidified incubator (5% CO_2_) for 6 h. Cells were washed twice with ice cold PBS containing 1mg/ml heparin, followed by one wash with trypan blue and two washes with ice cold PBS. Cells were trypsinised, followed by centrifugation at 700 g for 5 min at 4°C. Cell pellet was resuspended in ice cold PBS and FACS analysis was done in BD Accuri instrument. 10000 counts per sample was analyzed using BD Accuri C6 software.

### Quantitative real time PCR for miR-21

Cells maintained in growth medium (DMEM high-glucose, 10% FBS, without antibiotic and antimycotic) were passaged in 24-well plate (5 × 10^4^ cells/well), grown to 80% confluency and treated with different concentrations of the peptides. Cells were then incubated at 37°C humidified incubator (5% CO_2_) for 24 h. Total RNA was isolated from cells using TRizol® Reagent (Invitrogen). Expressions of mature microRNAs were determined by QuantiMir kit. (SBI, catalog number RA660A-1). Primers used for qRT-PCR is listed below:

Forward primer: TAGCTTATCAGACTGATGTTGA

Reverse primer: QuantiMir universal reverse primer

The microRNA expression levels were detected using SYBR-green I PCR master mix (Applied biosystems) on Roche Lightcycler LC 480 and normalized with respect to U1 as a reference gene. The Real Time data analysis was done by using 2(-Delta Delta C(T)) method (47).

### miRNA expression profiling

MCF-7 cells were cultured in 6-well plate (3 × 10^5^cells/well), grown to 80% confluency and treated with peptide in a final concentration of 1 μM. 24 h post treatment, total RNA was isolated using TRizol® Reagent (Invitrogen, USA). 1 μg of starting RNA was used to synthesize cDNA. Universal Reverse primer was provided by Human miRNome profiler kit (SBI). MiRNA expression profiling was done in pre-formatted qPCR array format as provided by System Biosciences (SBI).

### Western blot

Total protein was isolated from treated and untreated MCF-7 cells using cell lysis RIPA buffer (Pierce Chemical Co., Rockland, IL). Four LNA modified anti-miR-21 (100 nM) (Anti-miR-21 sequence: TCAACATCAGTCTGATAAGCTA (residues underlined are LNA modified)) transfected sample was also taken as positive control. Protein concentration was estimated using BCA™ protein assay (Pierce Chemical Co., Rockland, IL). An equivalent amount of protein from each treatment was resolved on 12% SDS-PAGE. Following transfer to nitrocellulose membrane and blocking with 5% non-fat milk, the blot was incubated with primary antibody (1:1000) specific for PDCD4 protein and PTEN(abcam). After washing the blot with 1X TBST, it was probed with alkaline phosphatase conjugated secondary antibody (1:10000 dilutions)(abcam). The blot was developed using AP developing solution (Sigma) followed by densitometric analysis (ImageJ software, NIH).

### Detection of proliferation using Immunofluorescence

Cells were seeded on square coverslips in six-well plates and treated with peptide and mutant at 1 μM for 48 h. Subsequently, cells were fixed with 2% paraformaldehyde for 20 min at room temperature. For permealization, 0.5% Triton X-100 prepared in phosphate-buffered saline (PBS) was applied for 15 min at room temperature, followed by three washes with PBS for 5 min each. Nonspecific binding was blocked by incubation with 5% bovine serum albumin (BSA) in PBS for 30 min at room temperature. The cells were treated with primary anti-Ki67 antibody (1:1000, Abcam) and incubated overnight at 4°C. To visualize Ki67, cells were incubated with Alexa Fluor 488-labeled secondary antibody (1:250 dilutions, Invitrogen) for 45 min at room temperature. All antibodies and staining reagents were diluted in 5% BSA-PBS. Coverslips were mounted with ProLong Gold antifade reagent with DAPI (Life Technologies). Images were taken using a Zeiss LSM 510 Meta confocal microscope at 63X.

### Apoptosis detection by TUNEL assay

MCF-7 cells were seeded on poly-lysine coated coverslips in 6-well plates (3 × 10^5^ cells/well) and treated with peptide and mutant at a final concentration of 1 μM and grown for 48 h. Next, the cells were washed twice with 1X PBS, and fixed in 4% paraformaldehyde for 20 min at room temperature. The fixed cells were permeabilized with 0.2% Triton X-100 for 5 min at room temperature, followed by washing with 1X PBS. To determine the effect of peptide treatment on apoptosis, we performed the apoptosis assay using the DeadEnd™ Colorimetric TUNEL System kit (Promega) following the instructions of the manufacturer. Briefly, the cells were incubated with TUNEL reaction mixture (biotinylated nucleotide mix) for 60 min at 37°C in a humidified chamber. Negative control was prepared and was treated with the TUNEL label reaction mixture (without Terminal deoxynucleotidyl transferase enzyme). Endogenous peroxidase activity was quenched using 0.3% H_2_O_2_ for 5 min at room temperature. The cells were treated with streptavidin-HRP (1:500) and stained with DAB components. The cells were washed with de-ionized water several times before imaging.

### Invasion and migration assay

Cancer cell invasion and migration assay was done using CytoSelect cell migration and Invasion assay kit (Cell Biolabs, Inc.) using manufacturer's protocol. Briefly, 24 h after treatment, cells were counted and placed on control inserts or Matrigel inserts at 10^5^ cells/ml in serum-free medium and were allowed to migrate for 24 h at 37°C. Cells were removed from the top of the inserts and cells that migrated/invaded though the polycarbonate basement membrane were fixed, stained and images were taken in light microscope. Next, the stained basement membrane was extracted and quantified at optical density of 560 nm.

### Cell viability assay

MCF-7 cells were seeded in 96 well-plates (8000 cells/well), grown to 80% confluency and then treated with peptides at different concentrations ranging from 0 to 1 μM. After 24 and 48 h of growth, cells were treated with 1 mg/ml 3-(4, 5-dimethylthiazol-2-yl)-2, 5-diphenyltetrazolium bromide (Sigma-Aldrich) and incubated for 4 h at 37°C in CO_2_ incubator. After incubation, the medium was removed and formazan crystals were dissolved in 200 μl DMSO solution. Sample absorbance was measured at 570 nm with a reference wavelength of 630 nm. Data were normalized with untreated control set.

## RESULTS

### Phage display against pre-miR-21

To find out selective peptides against pre-miR-21, we have used phage display. A schematic presentation of the workflow has been summarized in supporting information Scheme S1.

Pre-miR-21 was *in vitro* transcribed followed by biotinylation at the 3′ end. Solution phage panning was carried out with streptavidin coated magnetic beads. After doing negative selection against streptavidin and tubes, four rounds of panning were carried out with increasing stringency. To achieve selectivity against pre-miR-21, non biotinylated pre-miR-27a (for the first two rounds of panning) and HEK293T total cellular RNA, where miR-21 expression is reported to be very low (48,49), (for the last two rounds of panning) were pre-incubated to the phage pool, followed by panning against pre-miR-21. Deep sequencing of the amplified library was done after fourth round of panning. Out of 12261 reads, 9513 reads correspond to a common peptide motif ‘ALWPPNLHAWVP’ and the others were found be the same motif with one or two amino acid substitutions. Traditional colony based sequencing was also carried out. 20 colonies against pre-miR-21 were sequenced after fourth panning and all of them gave the same motif of ‘ALWPPNLHAWVP’. This indicated that ALWPPNLHAWVP peptide can be the potential binder of pre-miR-21.

### *In vitro* binding to pre-miR-21

**Figure 1.**
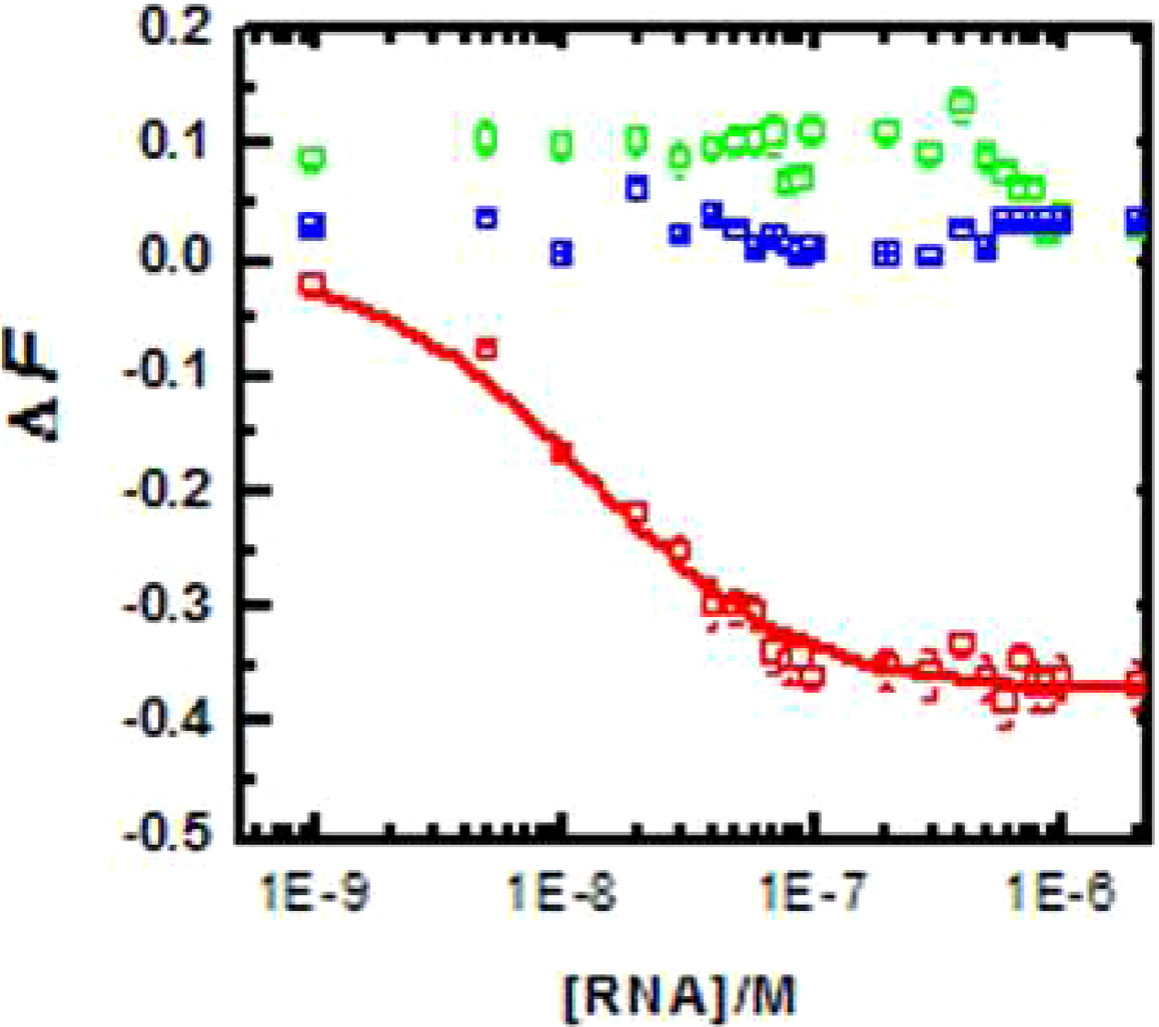
Binding analysis of peptide with pre-miR-21. Binding isotherm of wild-type peptide (red), reverse mutant (blue) and scrambled mutant (green) with pre-miR-21.

Next we wanted to explore the binding interaction between the ALWPPNLHAWVP peptide and pre-miR-21. Along this line we have taken two control peptides: (i) reverse mutant: PVWAHLNPPWLA and (ii) scrambled mutant: LPPAPWNWAHVL. All the peptides have the same amino acid composition, while the sequences are different and hence will allow us to examine sequence specific interaction. All the peptides contain two tryptophan residues; allowing us to look for intrinsic fluorescence change in the presence of pre-miR-21. In the presence of pre-miR-21, we found a decrease in fluorescence for the wild-type peptide, indicating strong interaction. The mutant peptides did not show significant change. Quantitative analysis of fluorescence titration (binding isotherm) showed strong binding of pre-miR-21 to wild-type peptide with dissociation constant 12.7 nM, while the mutants showed weak binding (Figure 1). This clearly indicated that the selected peptide binds to pre-miR-21 in a sequence-specific manner.

### Identification of the binding pocket of peptide to pre-miR-21

Provided the strong binding of the peptide to pre-miR-21 it was interesting to look for the binding pocket in pre-miR-21.

**Figure 2.**
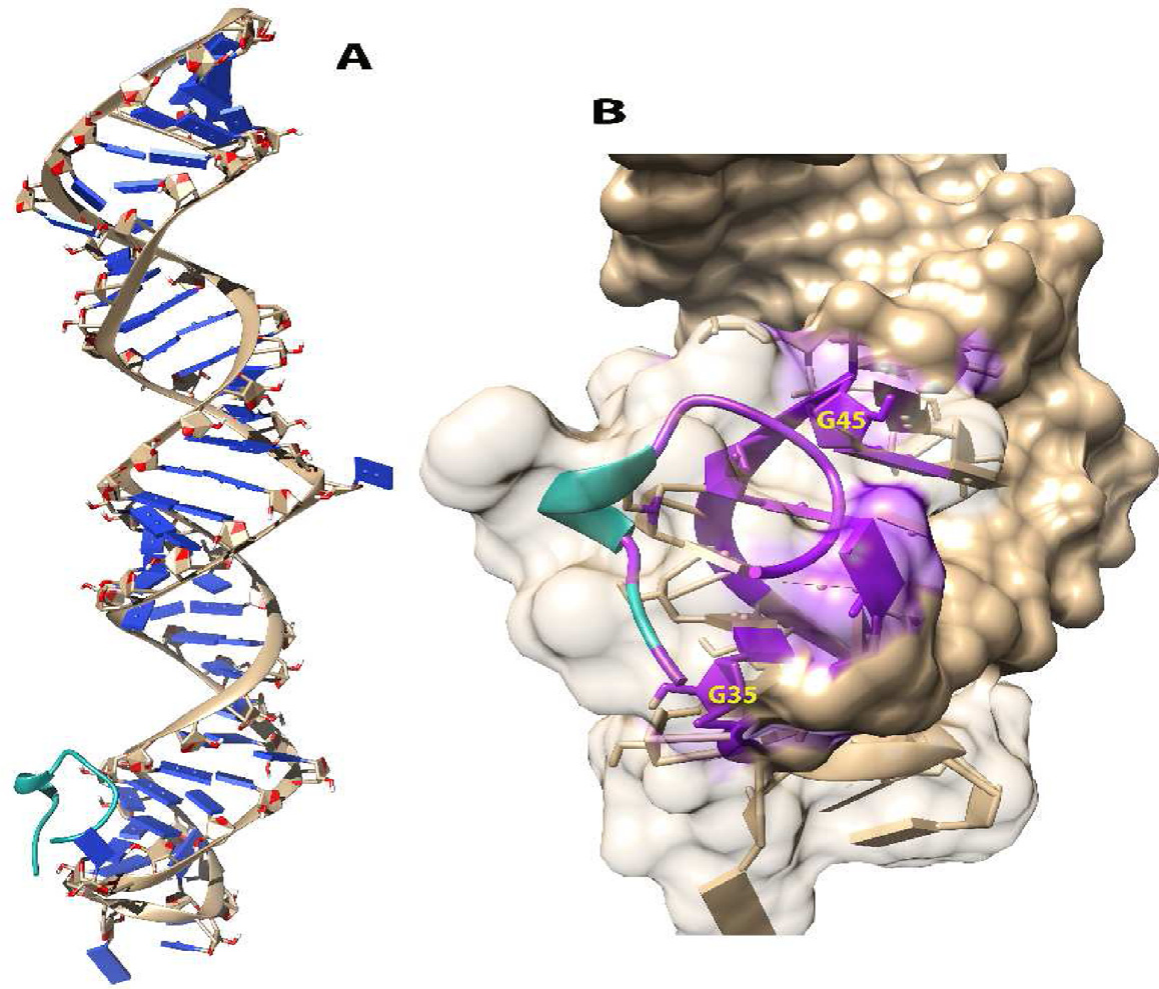
Manually picked model (Rank 4) having ZDock Score of 1267.761. (**A**) Docked model of peptide (coloured in green) bound to the pre-miR-21 (**B**) Magnified view of the docked structure showing the solvent accessible surface area (SASA) and contacts (coloured in purple) between the peptide and pre-miR-21. The SASA and contacts were calculated using Chimera.

An in-silico approach was used to predict and study the binding pose of the peptide to the miRNA. The first step involved the prediction of the secondary and tertiary structure of the pre-miR using mc-fold mc-sym pipeline. The energy-minimized model was obtained using the method's scoring function, which calculates the base pairing energy contribution by reducing the nucleotides into cyclic motifs. PEP-FOLD web server was used for the peptide structure prediction. PEP-FOLD predicts structure based on concept of structural alphabets. The best peptide model with the highest GDT score (0.732) was chosen for an all atomistic molecular dynamic simulation to check for conformational stability using GROMACS 4.6.1. A simulation of 100 ns revealed that the central residues were found to markedly more stable than the flanking region. Also the structure of the model remained stable post 20 ns (Supplementary Figure S1). This stabilized structure of the peptide was then used along with the modeled pre-miR for the docking study. For docking, we used ZDock, an FFT algorithm, which searches the rotational space to give a shape complimentary score. The method gave an ensemble of 99 models (Supplementary Figure S2), which were ranked to the above mentioned ZDock score. To further critically assess the prediction; the top 20 models were then manually studied to evaluate them based on the interactions between the peptide and pre-miR-21. The best model was chosen from this list; which had a high ZDock score (1267.761) as well as high intra-model residue contacts (Figure 2). The model chosen had interactions between residues (1–6, 10, 12) of the peptide and residues (43–46 and 35–32) of the pre-miRNA.

**Figure 3.**
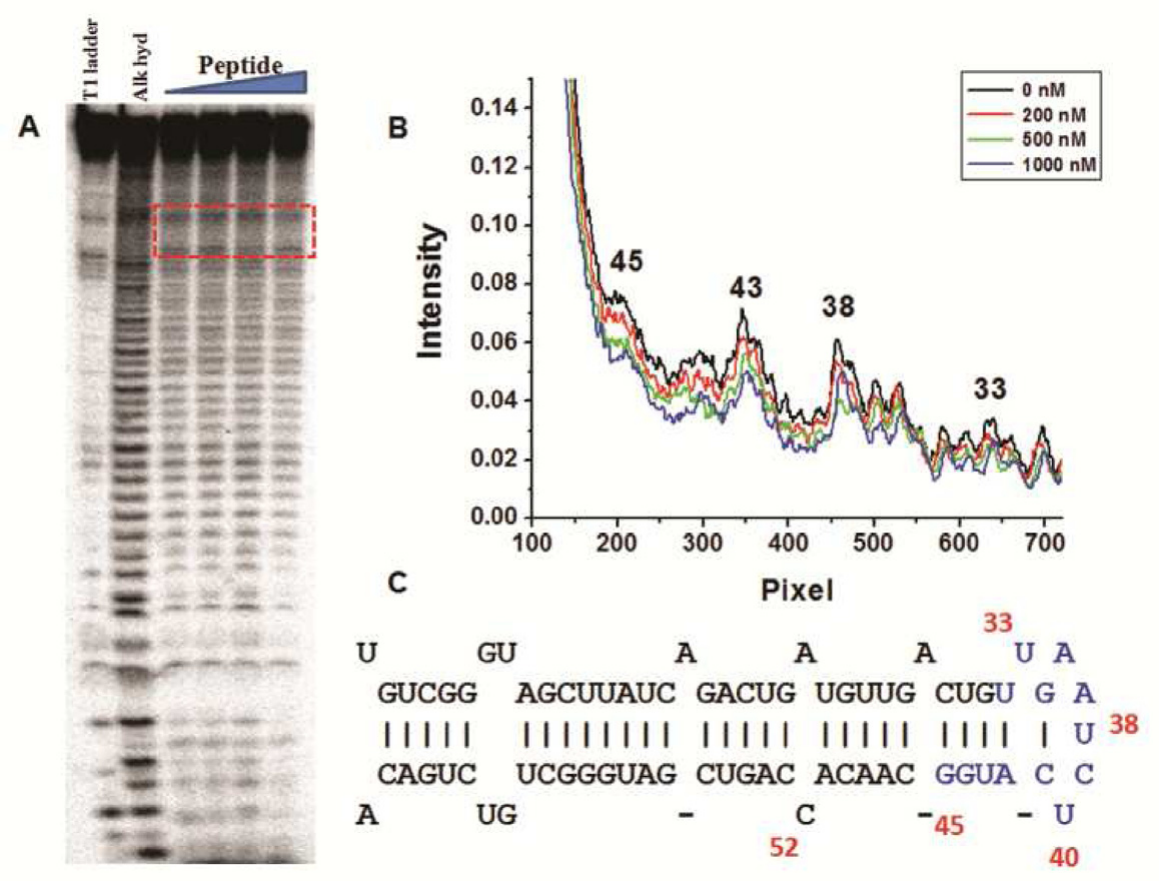
S1 nuclease probing of pre-miR-21 and peptide interaction. (**A**) Cleavage pattern of uncomplexed pre-miR-21(lane 3) and pre-miR-21 complexed with increasing concentrations of peptide (lanes 4 to 6). Lanes 1 and 2 represent the T1 digestion ladder and alkaline hydrolysis ladder, respectively. (**B**) Representative plot of the intensity of bands from nucleotides 31–45 for uncomplexed premiR-21 (black line), pre-miR-21 complexed with 200 nM, 500 nM and 1 μM peptide (red, green and blue line). (**C**) The sites of protection mapped to the pre-miR-21 structure(colored in blue, numbered in red).

To validate the *in-silico* result further, we did enzymatic footprinting of pre-miR-21 in the presence of increasing concentration of the peptide (0–1 μM). S1 nuclease footprinting was done to figure out the binding pocket. S1 nuclease cleaves single stranded and unconstrained nucleic acid endonucleolytically. A closer inspection indicated that residues 33–45 showed protection in S1 nuclease footprinting (Figure 3 and Supplementary Figure S3). This reduced cleavage in the presence of increasing concentrations of peptide indicated the binding pocket lies near the stem-loop junction of pre-miR-21 (Figure 3).

### Exploring inhibitory role of the peptide

**Figure 4.**
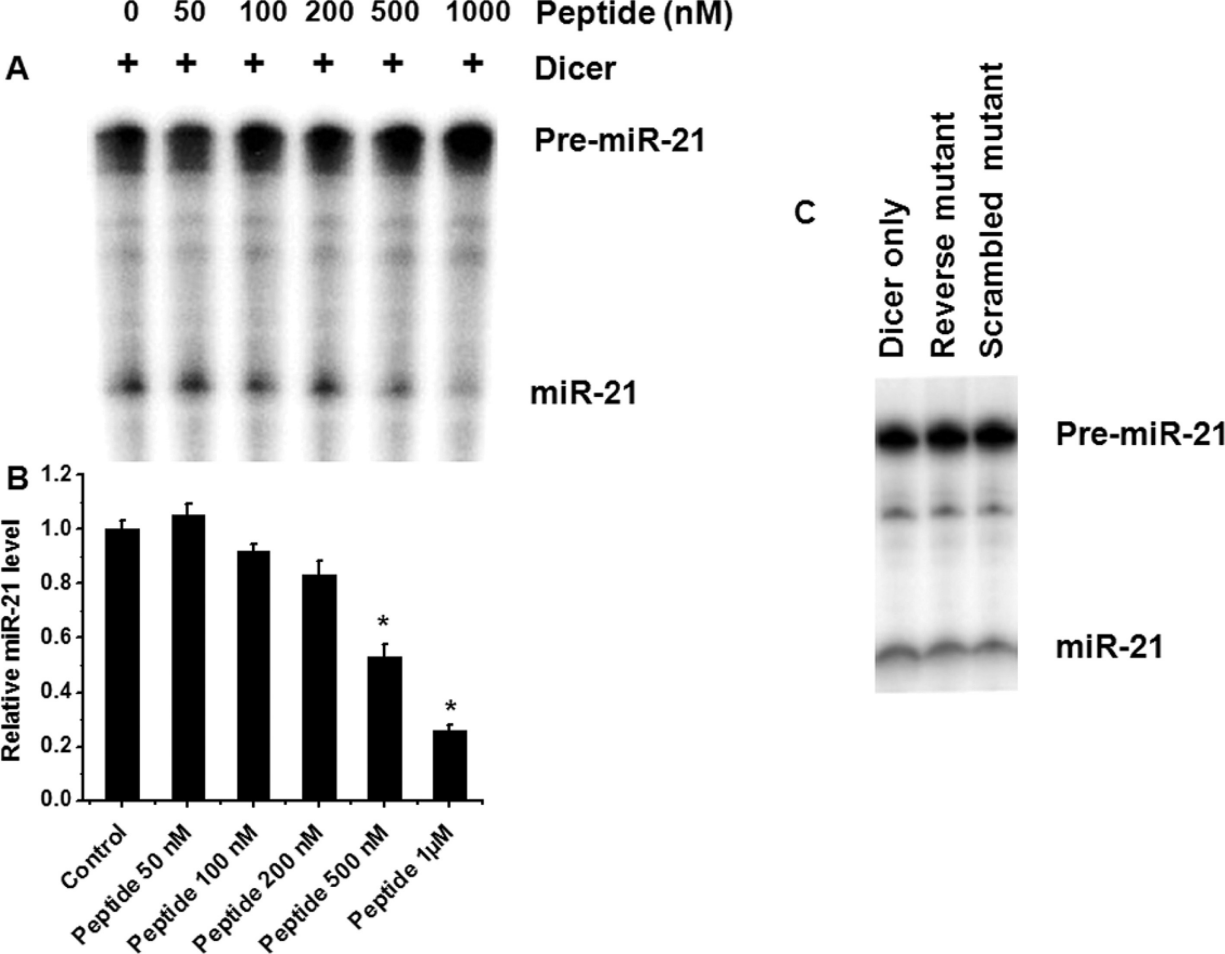
Dicer blocking assay. (**A**) Dicer blocking assay with wild-type peptide (lane 1: pre-miR-21 with Dicer, lanes 2 to 6: pre-miR-21 with Dicer in the presence of increasing concentrations of peptide). (**B**) Densitometric analysis of miRNA levels for the mentioned above assays showing a decrease in miR-21 formation with increasing concentration of peptide. (**C**) Dicer blocking assay in the presence of the two mutant peptides did not show any effect in miR-21 maturation. Error bars represent ±SD, calculated from three independent experiments.*, *P* < 0.01 (Student's *t*-test).

Given that the peptide binding pocket is near the stem loop junction, a portion where Dicer cleaves to form mature miRNA, it was interesting to ask whether the peptide binding can hinder Dicer processing and thus inhibit mature miRNA formation. To this end, we did Dicer cleavage assay in the presence of increasing concentration of the peptide. A significant decrease in mature miR-21 level was found in the presence of increasing concentration of peptide (0–1 μM), while the mutant peptides were unable to show any significant change in mature miR-21 formation (Figure 4). This clearly indicates that the peptide can inhibit mature miR-21 formation *in vitro* by binding to the stem-loop junction of pre-miR-21 and thereby blocking Dicer processing.

### Probing the importance of single nucleotide bulges near the terminal loop of pre-miR-21; in peptide binding and hence inhibitory function

We wanted to probe the importance of the bulges near the terminal loop (as revealed by enzymatic probing) in the binding interaction of peptide and pre-miR-21. To this end, we used a mutant pre-miR-21 where the bulge is absent (Supplementary Figure S4). Fluorescence titration study showed very weak binding of the peptide to this mutant pre-miR-21 (Supplementary Figure S5). *In-vitro* enzymatic footprinting did not show any protection as previously observed in the presence of peptide (Supplementary Figure S6). Dicer blocking assay did not show any effect in miR-21 maturation in the presence of peptide (Supplementary Figure S7). Collectively the evidence suggests that the bulges are important structural determinants for peptide binding and hence inhibition of Dicer processing. We have also checked the binding of the peptide to two other structurally different oncogenic pre-miRNAs (pre-miR-95 and pre-miR-103) and did not find any significant binding interaction (Supplementary Figure S5). This suggested selectivity of the peptide to pre-miR-21.

### Assessing miR-21 inhibitory efficiency inside the cell

As the peptide contains three proline residues, we reasoned that the peptide may have cell penetrating properties. To check the cell penetrating property, we FAM conjugated the peptides and monitored their uptake by flow cytometry. Flow cytometry analysis clearly indicated cell penetrating potential of the wild-type peptide, as well as the scrambled and reverset peptides (Supplementary Figure S8).

**Figure 5.**
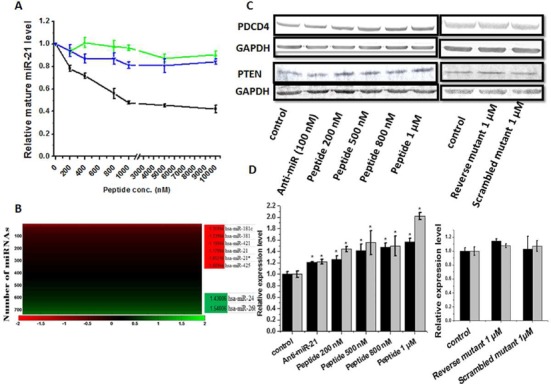
Inhibitory potential inside cell. (**A**) Relative levels of mature miR-21 shown at increasing concentrations of wild-type (black) and mutant peptides (green and blue) treatment (200 nM to 10 μM). (**B**) miRnome wide change in miRNA expression level (log_2_ fold change) upon 1 μM peptide treatment. (**C**) Western blot detection of PDCD4 and PTEN shows increased levels upon peptide treatment, while mutants were ineffective. Anti-miR-21 (100 nM) was taken as positive control. (**D**) Relative fold-change analysis (black bar for PDCD4 and gray bar for PTEN). Error bars represent ±SD, calculated from three independent experiments.*, *P* < 0.01 (Student's *t*-test).

To evaluate the efficacy of the peptide inhibitor inside cell, we checked mature miR-21 expression using qRT-PCR in MCF-7 cells (where endogenous miR-21 is reported to be over expressed). A dose dependent (0–10 μM) inhibition of miR-21 level was found after 24 h of treatment with saturation at 1 μM of peptide, confirming its inhibitory potential inside the cell. The scrambled and reverse peptides were totally ineffective in down regulating miR-21 (Figure 5A).

To check the selectivity of the peptide against miR-21 inside the cell, qRT-PCR based miRNome profiling was done in MCF-7 cells using SBI miRNome miRNA profiler. In MCF-7 significant expression level of 762 miRNAs were detected. After 24 h of treatment of 1 μM peptide, 13 miRNAs were found to be down regulated and 20 miRNAs to be upregulated (2-fold expression changes was the cut-off). Literature survey indicated that out of those 33 miRNAs, 24 miRNAs were discovered via high throughput sequencing studies and are not functionally annotated. With the rapid advancement of the sequencing technology, numerous novel miRNAs are being constantly deposited in the miRBase database, but many of them are turning out to be not bona fide miRNAs and are by product of annotation assignment error (50,51). Thus, we removed those 24 low confidence miRNAs and out of the resultant 9 miRNAs, 6 were down-regulated and 2 were up-regulated (Figure 5B). Both miR-21 and miR-21* was found to be significantly down-regulated in this miRNome profile also. This result clearly indicates that the peptide is very selective toward miR-21.

Next, we wanted to assess the impact of the peptide on miR-21 functional targets, on whether it can antagonize miR-21 function. We checked the expression level of two miR-21 targets, PDCD4 and PTEN. In the presence of increasing concentration of peptide (0–1 μM), we found increase in both PDCD4 and PTEN in MCF-7. The two mutants were not effective (Figure 5C and D). We also checked the expression of PDCD4 in the presence of peptide (1 μM) in HEK293T cell line where the miR-21 expression is low (48,49) and we did not find any significant change compared to the untreated control (Supplementary Figure S9A). Along the same line, we over-expressed wild-type pre-miR-21 and mutant pre-miR-21 in HEK293T cell line and then checked the effect of the peptide. Over-expression of either of the two pre-miR-21 reduced the level of PDCD4 compared to the control, as in the both the cases mature miR-21 was formed. Peptide was able to restore the level of PDCD4 back to the control, but was ineffective in case of mutant pre-miR-21, due to its inability to bind to mutant pre-miR-21 (Supplementary Figure S9B and S9C). Taken together, the peptide inhibits miR-21 formation and thereby up-regulates its target proteins.

### Effect of peptide on cancer cell proliferation and apoptosis

**Figure 6.**
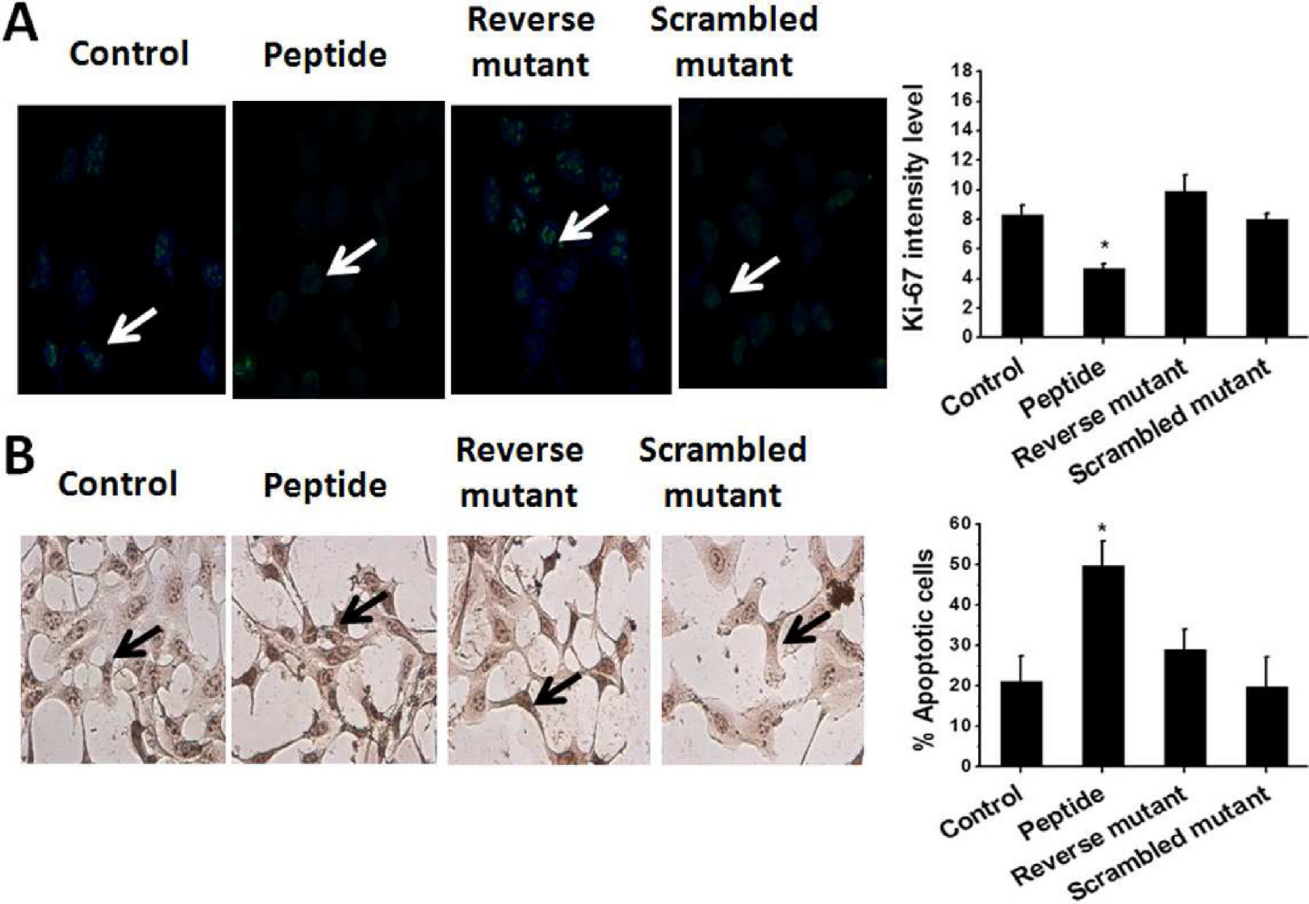
Impact of peptide on cell proliferation and apoptosis. (**A**) Peptide treated cells revealed lower level of Ki-67 marker, indicating inhibition of cell proliferation. The mutants were ineffective. (**B**) Peptide treated cells revealed increased number of tunel positive apoptotic cells (∼30%), while the mutants did not show significant effect. Error bars represent ±SD*, *P* < 0.01 (Student's *t*-test).

Proliferation is a hallmark of tumorigenesis. PDCD4, one of the important targets of miR-21, regulate translation by directly interacting with translation initiation factor eIF4A and blocking eIF4G helicase activity and thereby causes decrease in protein synthesis, inhibition of proliferation and increase in apoptosis (52–54). Thus, silencing of miR-21 suppresses tumor cell proliferation and increases apoptosis. We went on to check the potential of miR-21 inhibitor peptide to modulate cell proliferation and apoptosis. Immunostaining with anti-Ki-67 cells showed fewer Ki-67 positive cells in peptide treated condition, compared to the untreated control (Figure 6A) while the mutants did not show the effect. We did TUNEL assay to check for apoptosis induced by the peptide and found that there was around 30% increase of apoptosis in the presence of the peptide inhibitor (Figure 6B). Along the same line, we did MTT assay to look for cell viability in the presence of different concentrations (0–1 μM) of peptide. Around 20% cell death was found after 48 h of treatment, which is probably due to increased apoptosis (Supplementary Figure S10). There was no significant change in cell viability after 24 h of treatment (Supplementary Figure S10). We also checked the effect of the peptide on cellular apoptosis in HEK293T cell line where miR-21 expression is low (48,49) and no significant increase in apoptosis was found compared to untreated control (Supplementary Figure S11). Taken together, our data indicate that the peptide is potent to suppress tumor cell proliferation and survival via miR-21 downregulation.

### Effect of peptide on cell invasion and migration

**Figure 7.**
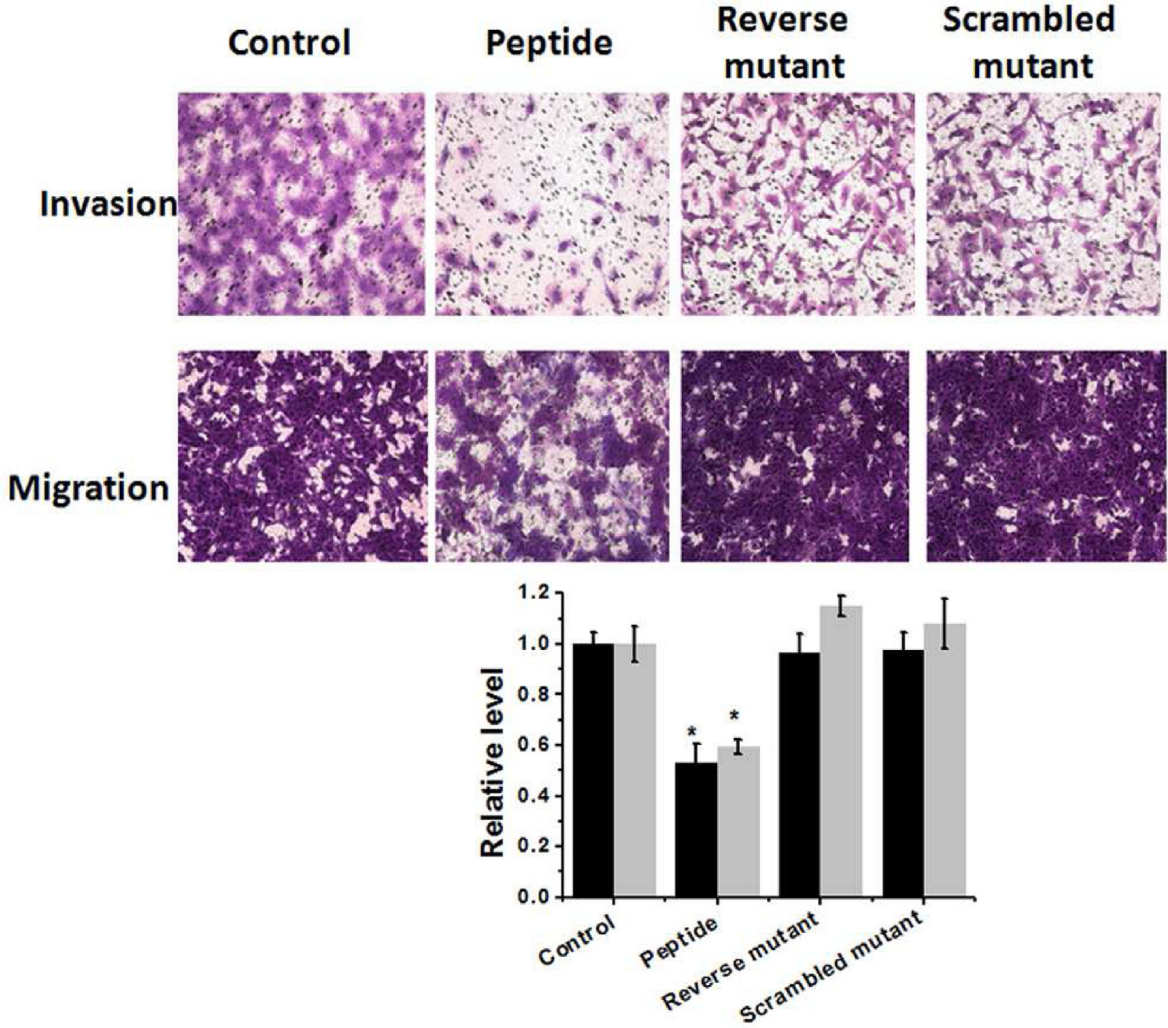
Impact of peptide on MCF-7 cell invasion and migration. (**A**) Peptide treated cells revealed reduced cell invasion and migration, while mutants were ineffective. (**B**) Quantitative analysis showed ∼50% reduction in cell invasion (black bar) and ∼30% reduction cell migration (gray bar) upon peptide treatment. Error bars represent ±SD, calculated from three independent experiments.*, *P* < 0.01 (Student's *t*-test).

Tumor cell invasion and migration are crucial steps of metastasis and miR-21 helps in cell migration and invasion in different kinds of cancer (including breast cancer) by targeting tumor suppressors PDCD4, PTEN, TMP1 and maspin (55). Down-regulation of miR-21 was shown to inhibit cell migration and invasion both in cell and in vivo (36,55). To assess the impact of the peptide inhibitor on cell migration, we performed scratch wound assay. In this assay a linear scratch was made in the cell layer and cell's ability to heal the scratch (because of its migration potential) was assessed. The peptide was found to be a potent inhibitor of MCF-7 migration (Supplementary Figure S12). We also wanted to check the effect of this peptide in a more metastatic cell line MDMB231, where miR-21 is also over-expressed. We checked the endogenous level of miR-21 in MDMB231 cell line in the presence of peptide (1 μM) and found down-regulation of miR-21 upon peptide treatment (Supplementary Figure S13). Scratch assay in MDMB231 showed similar results (Supplementary Figure S12). We also did transwell assay to monitor cell invasion and migration. As shown in Figure 7 and Supplementary Figure S14, we found a significant decrease in cell invasion and migration (both in MCF-7 and MDMB231 cell lines) in peptide treated group compared to untreated control, while the mutant was ineffective. The peptide was ineffective in reducing cell invasion and migration in low miR-21 condition, HEK293T cell line (Supplementary Figure S15). These results clearly indicate that the peptide is capable of inhibiting cancer cell invasion and migration by antagonizing miR-21.

## DISCUSSION

Distinct groups of miRNAs are reported to be up-regulated or down-regulated in different types of cancer and are associated with disease progression and maintenance. As a single miRNA controls multiple genes, miRNA modulation based therapeutics is emerging to be a potential new avenue to target multiple gene interaction networks and thereby target cancer with more efficacies. Given the drawbacks of oligonucleotide based therapeutics, recent efforts have been put forward to look for alternate strategies for modulation of miRNA. Different groups, including us, have reported small molecule inhibitors against miRNA. Though these small molecules were shown to have some selectivity against a particular miRNA, none of them were tested for their off-target effect in miRNome expression. With the recent improvement in peptide based therapeutics, because of its better specificity and hence less off target effect, it is worth to explore peptide based specific modulator of miRNA. Diaz *et al*. have previously shown the potential of peptoid to down regulate miR-21 *in vitro* (22).

In our study, using phage display we have identified a specific peptide against oncogenic miR-21. It inhibits mature miR-21 formation by binding to pre-miR-21 and thereby Dicer processing. The peptide is also effective in down regulating miR-21 in cells and hence antagonizing its function. It is specific to miR-21 inside the cell. The peptide was effective in inhibiting cancer cell invasion, migration and proliferation and activating apoptosis by modulating miR-21 targets, including PTEN and PDCD4. Taken together, this peptide and its peptidomimetic derivatives can be explored for its potential anti cancer activity in vivo and may further be extended to clinical studies.

## SUPPLEMENTARY DATA

Supplementary Data are available at NAR Online.

SUPPLEMENTARY DATA
