# Supplementary material for: Selective inhibition of miR-21 by phage display screened peptide
Source: Nucleic Acids Res. 2015 Mar 30;43(8):4342–52. doi: 10.1093/nar/gkv185 (PMC4417150; doi:10.1093/nar/gkv185)
Supplement: SUPPLEMENTARY DATA [file supp_43_8_4342__index.html]

Selective inhibition of miR-21 by phage display screened peptide — SUPPLEMENTARY DATA 

# Selective inhibition of miR-21 by phage display screened peptide

## SUPPLEMENTARY DATA

**Files in this Data Supplement:**

- SUPPLEMENTARY DATA
