# Supplementary material for: Selective inhibition of miR-21 by phage display screened peptide
Source: Nucleic Acids Res. 2015 Mar 30;43(8):4342–52. doi: 10.1093/nar/gkv185 (PMC4417150; doi:10.1093/nar/gkv185)
Supplement: SUPPLEMENTARY DATA [file supp_gkv185_nar-03368-y-2014-File002.doc]

**Supporting information**

**Selective inhibition of miR-21 by phage display screened peptide**

Debojit Bose^1^, Smita Nahar^1^, Manish Kumar Rai^1^, Arjun Ray^1^, Kausik Chakraborty^1^, and Souvik Maiti^1,2^

^1^ Proteomics and Structural Biology Unit, Institute of Genomics and Integrative Biology, CSIR. Mathura Road, Delhi 110020 (India)

^2^ National Chemical Laboratory, CSIR, Dr. Homi Bhabha Road, Pune 411008 (India)

To whom correspondence to be addressed: Souvik Maiti. Fax: +91-11- 2766-7471; Tel: +91-11- 2766-6156; E-mail: souvik@igib.res.in.

**Supporting Figures**


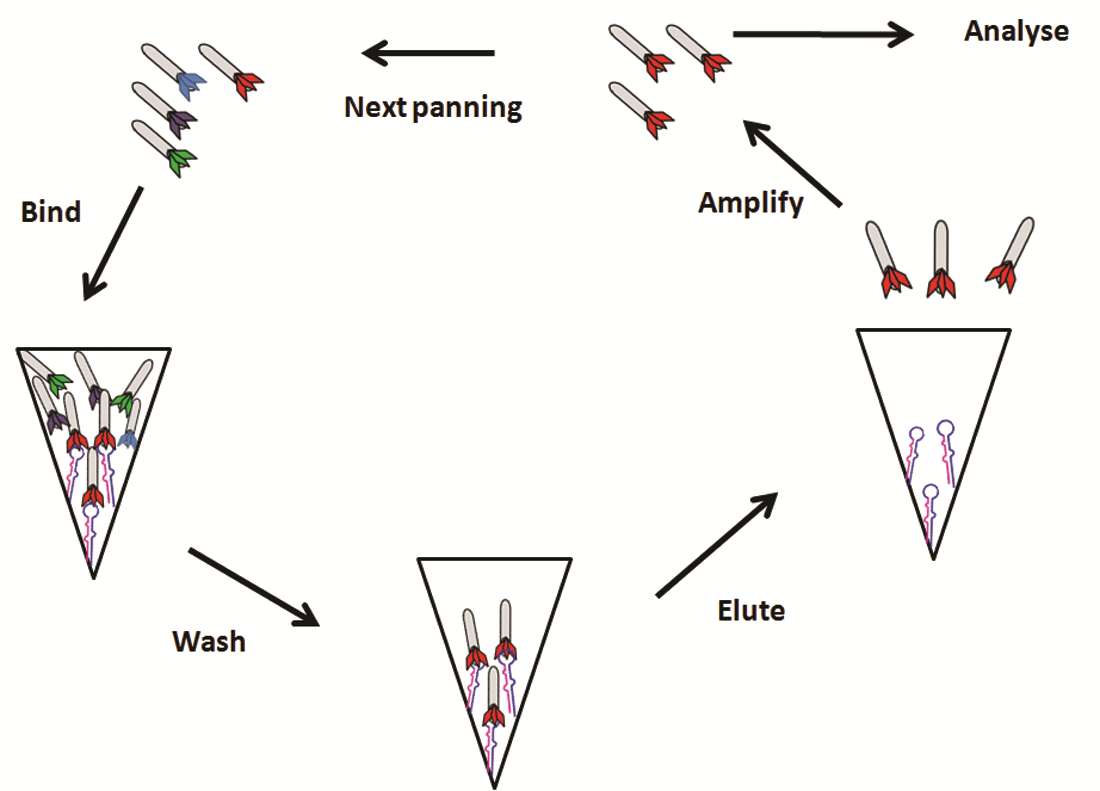


**Scheme S1.** Schematic representation of phage display.

The pre-miR-21 was immobilized in test tube and the phage pool was added to it. The non binding phages were washed off followed by the elution and amplification of bound phages. Amplified phages were used for next round of selection with increasing stringency. After panning the phage DNA was isolated and sequenced to figure out the corresponding peptide(s) that bind the pre-miR-21.


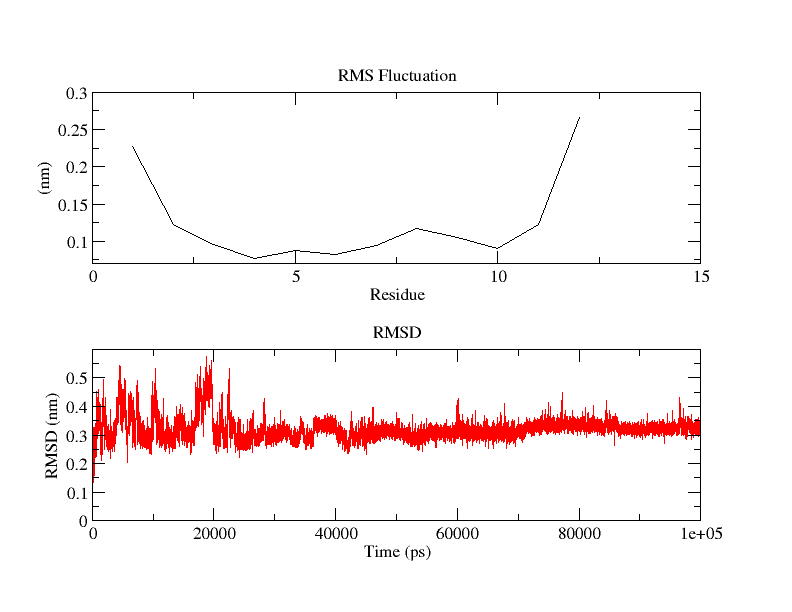


**Figure S1**. **MD simulation of peptide using GROMACS 4.6.3. for 100ns**. The panel above shows the RMS fluctuation across the simulation for each residue. Panel below shows the RMSD in nm for the peptide model over time (ps).


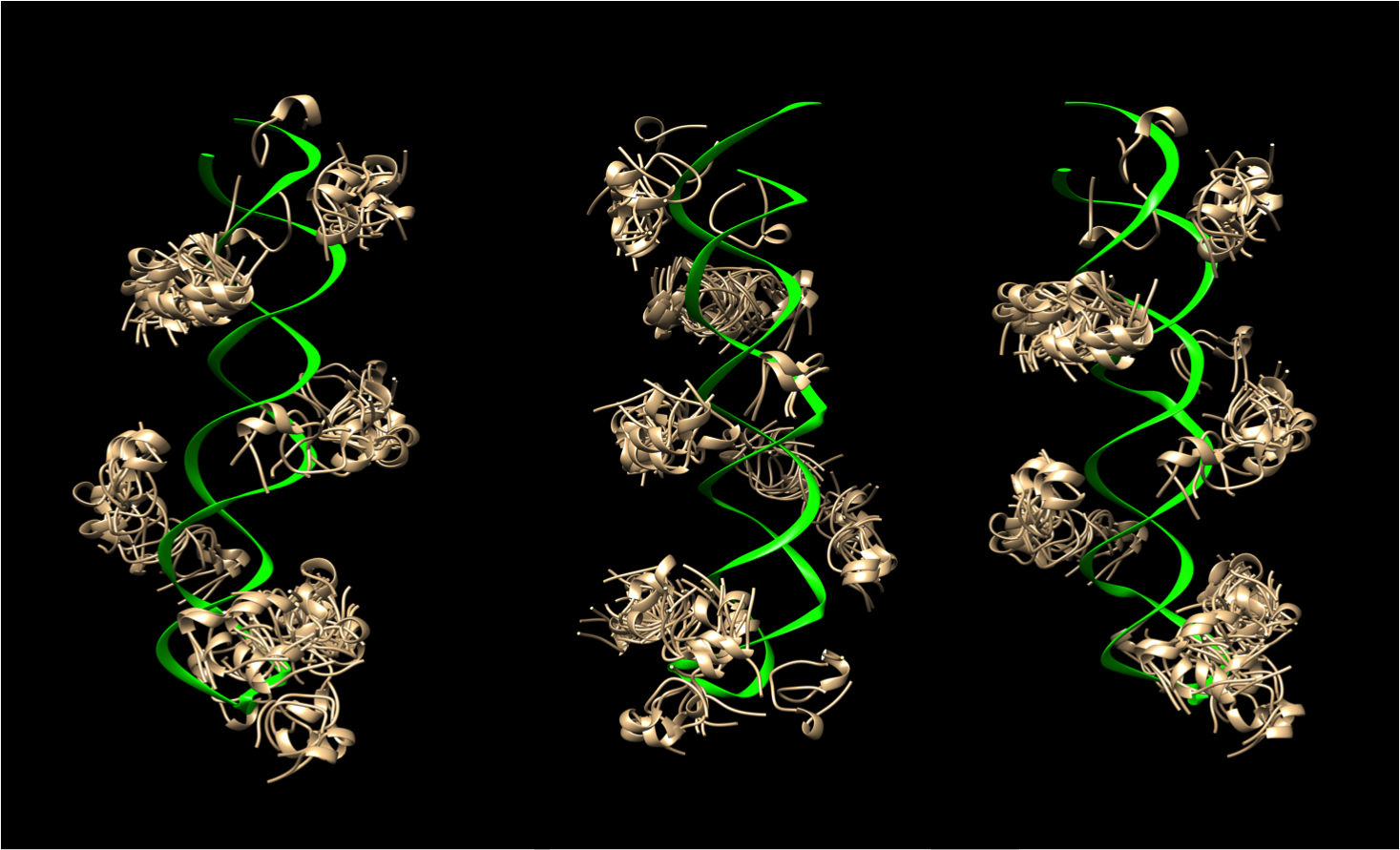


**Figure S2. 99 docking poses of peptide with miRNA using ZDock**. Figure were generated using Chimera


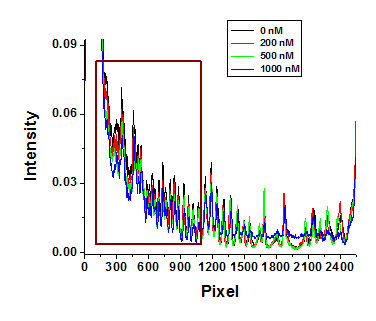


**Figure S3.** **S1Nuclease probing of pre-miR-21 and peptide interaction**. Representative graph plot of the intensity of bands for uncomplexed pre-miR-21(black line) and pre-miR-21 complexed with 200 nM (red line), 500 nM (green line) and 1 µM peptide (blue line). The portion highlighted, shows concentration dependent decrease in band intensity, which clearly indicates it as the binding pocket of the peptide.


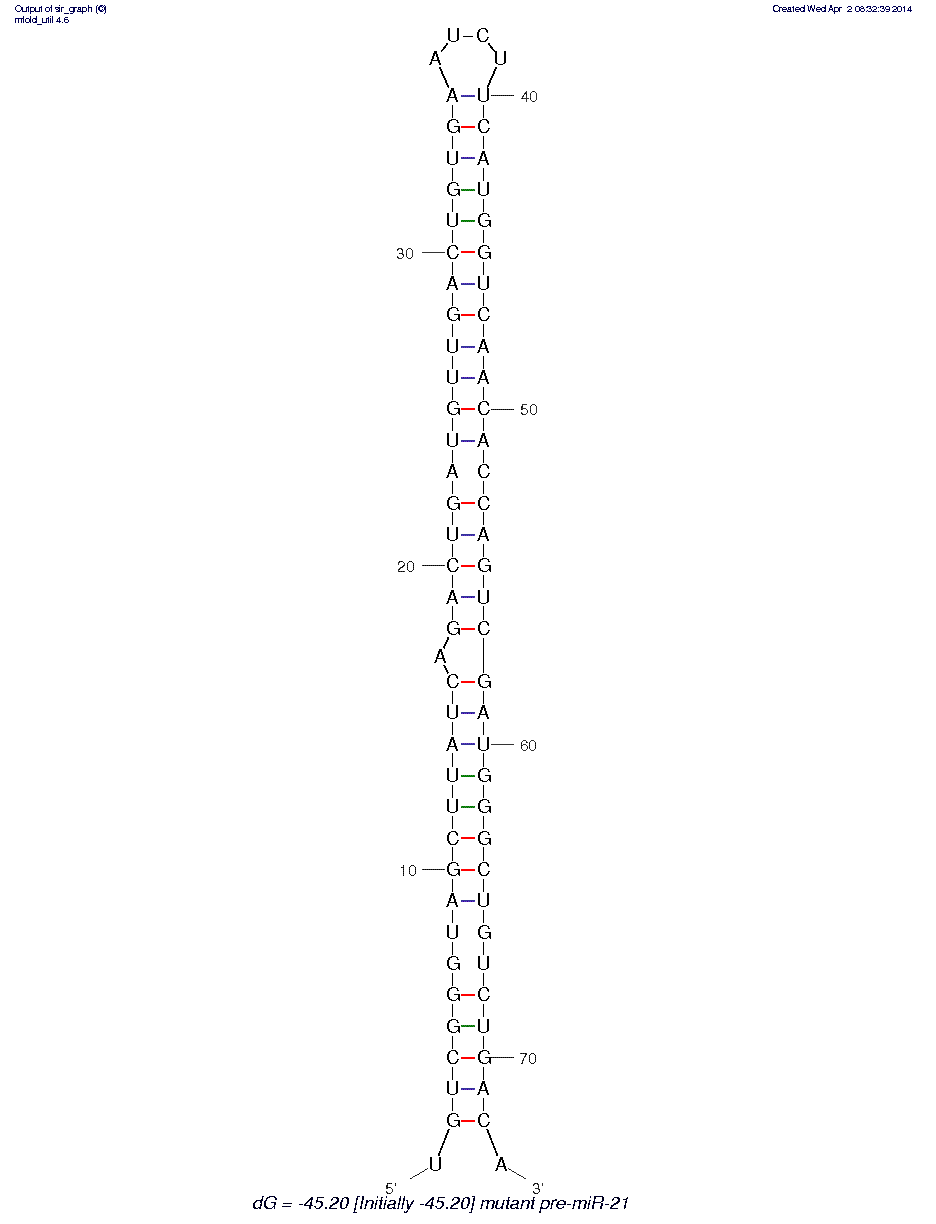


**Figure S4.** **Secondary structure of mutated pre-miR-21 revealed no bulge close to the terminal loop.**

**
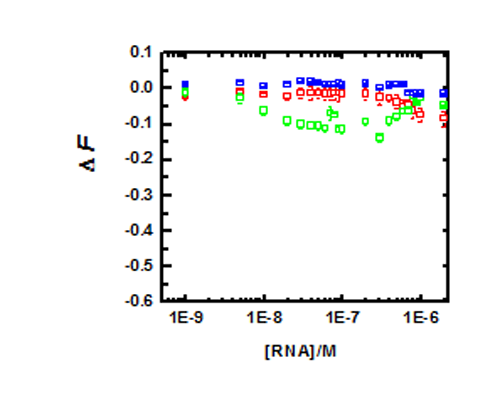
**

**Figure S5.** **Binding analysis of peptide with mutant pre-miR-21, pre-miR-95 and pre-miR-103.** Binding isotherm of wild type peptide with mutant pre-miR-21 (green), pre-miR-95 (blue) and pre-miR-103 (red).


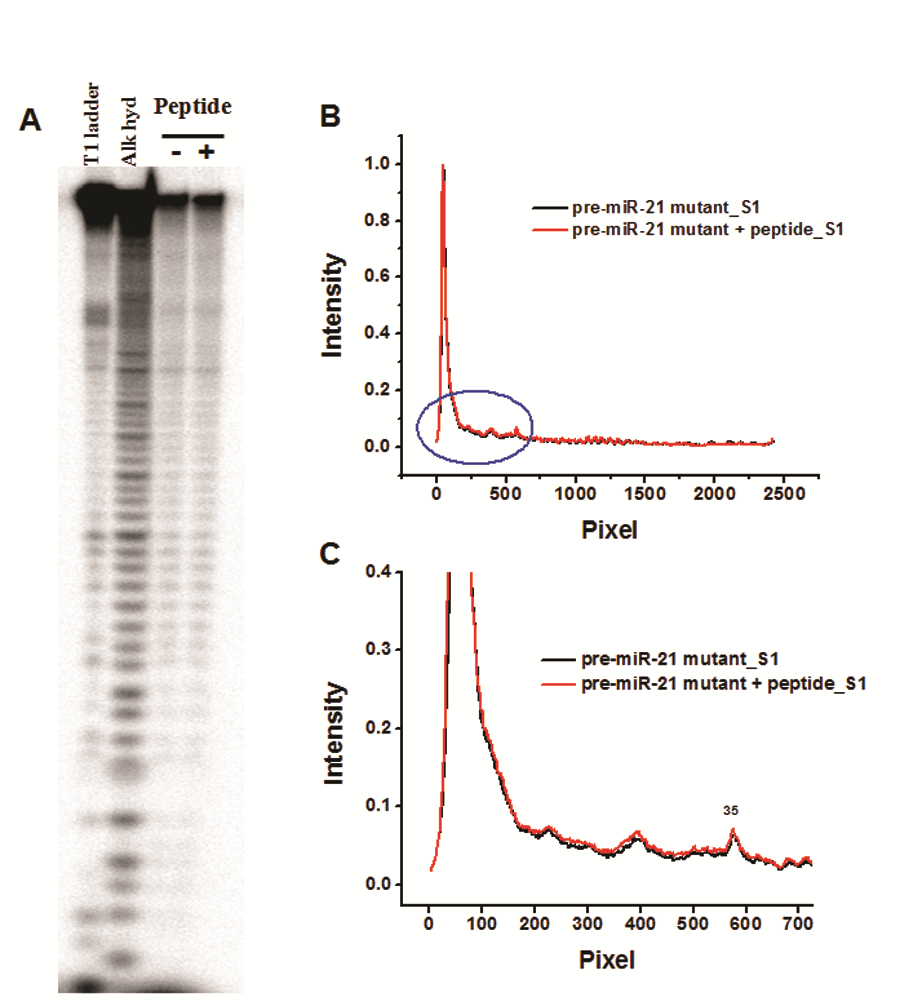


**Figure S6.** **S1Nuclease probing of mutated pre-miR-21 and peptide interaction.** (A) Cleavage pattern of uncomplexed mutant pre-miR-21(lane3) and pre-miR-21 complexed with 1 µM peptide (lane 4). Lane 1 and Lane 2 represent T1 digestion ladder and alkaline hydrolysis ladder respectively. (B) Representative graph plot of the intensity of bands for uncomplexed mutant pre-miR-21(black line), mutant pre-miR-21 complexed with 1 µM peptide (red line). (C) Zoom out portion indicated by a blue circle in B. Black and Red line perfectly overlap, indicating no protection and hence no binding to mutant pre-miR-21.


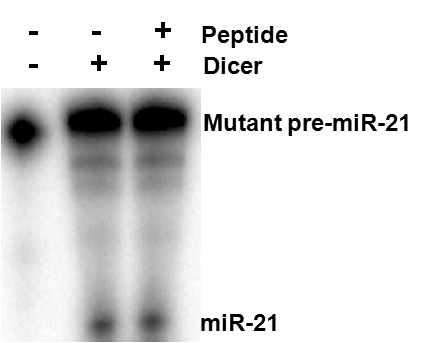


**Figure S7. Dicer blocking assay of mutant pre-miR-21.** Lane 1: mutant pre-miR-21, lane 2: mutant pre-miR-21 with Dicer, lanes 3: mutant pre-miR-21 with Dicer in the presence of peptide (1 µM) shows no significant change in the amount of miR-21 formed in presence of peptide.


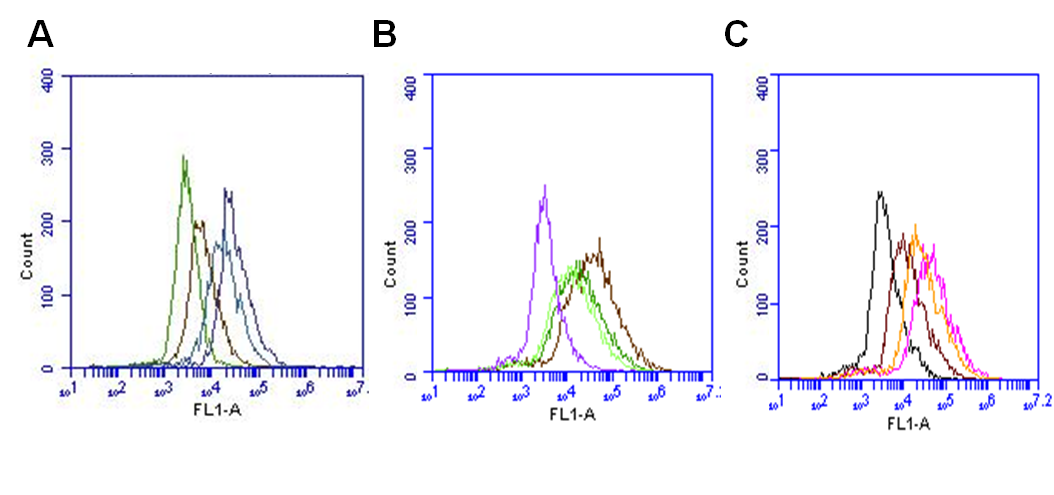


**Figure S8.** **Quantitative uptake of the peptide.** MCF-7 cells were incubated with increasing concentrations of the FAM-conjugated peptide. Peptide uptake in MCF7 cells was detected by FACS analysis of FAM-labeled cells. (A) Uptake of wild type peptide. green, brown, blue and purple indicate untreated, 1 µM peptide, 5 µM peptide and 10 µM peptide respectively. (B) Uptake of reverse mutant peptide. violet, light green, dark green and brown indicate untreated, 1 µM peptide, 5 µM peptide and 10 µM peptide respectively. (C) Uptake of scrambled mutant peptide. black, violet, orange and pink indicate untreated, 1 µM peptide, 5 µM peptide and 10 µM peptide respectively. The experiment was repeated in duplicate with comparable results. The graph shows one representative experiment. FL1-A indicates FAM log scale.


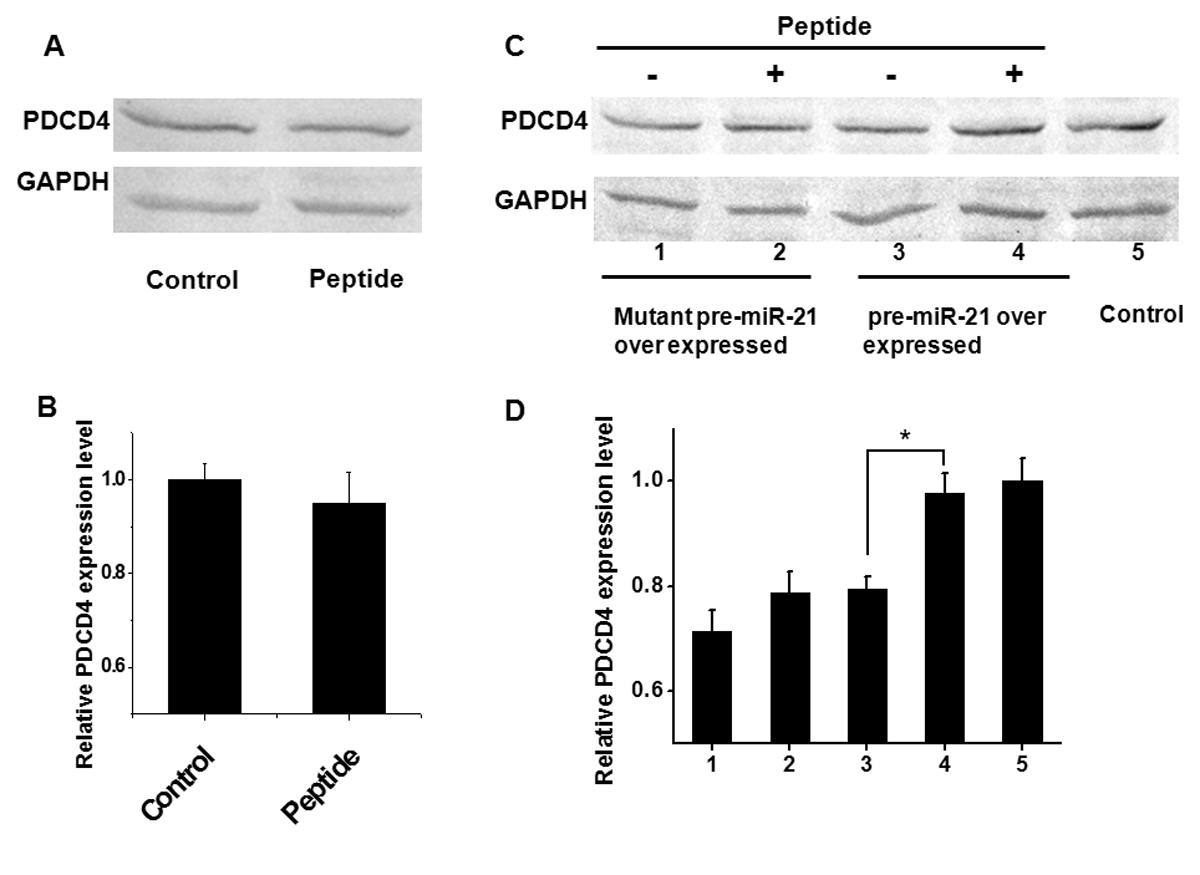


**Figure S9*.*** (A) Western Blot detection of PDCD4 level shows no significant change upon peptide treatment (1 µM) compared to untreated control. (B) Relative fold change analysis. (C) wild type pre-miR-21 overexpression caused reduction of PDCD4 level (lane 3) and peptide treatment showed restoration of PDCD4 level to untreated control (lane 4). Mutant pre-miR-21 overexpression reduced PDCD4 level (lane 1) and peptide was ineffective in restoring the level (lane2). (D) Relative fold change analysis.


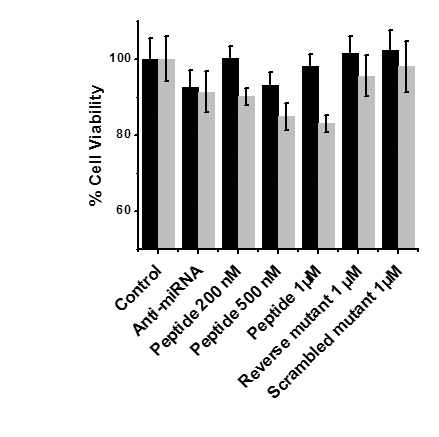


**Figure S10*.*** **Cell viability assay: Percentage of cell viability after treatment.** Data was normalized with untreated control set and Anti-miRNA (100 nM) was taken as a positive control. There was no cell death after 24 hrs (black bar) while there was ~20% cell death after 48 hrs (grey bar) upon peptide treatment. The mutant did not show any cell death even after 48 hrs.


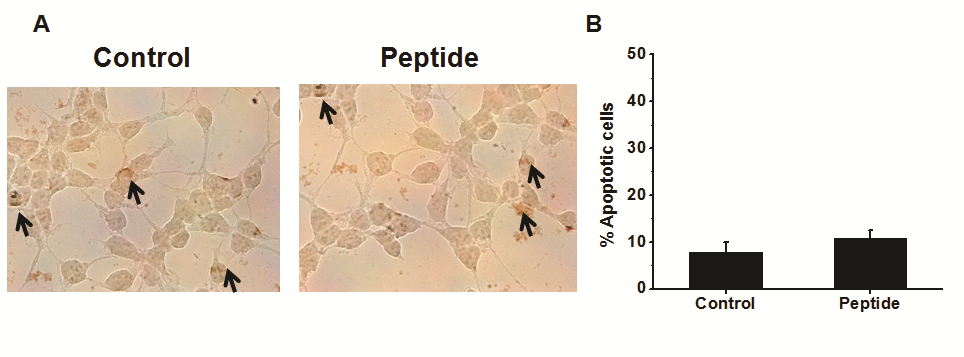


**Figure S11. Impact of peptide on apoptosis in HEK293T cells** (A) Peptide treated cells revealed no significant change in the number of tunel positive apoptotic cells, compared to untreated control. (B) Densitometric analysis. Error bars represent ± S.D.


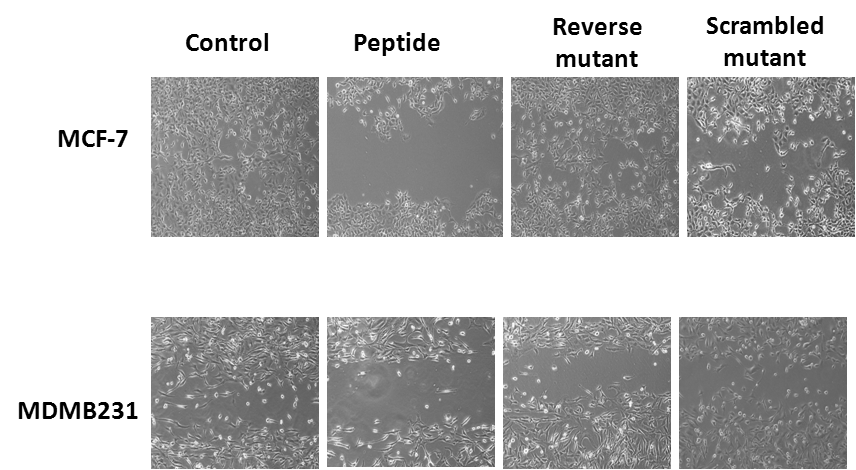


**Figure S12.** **Scratch wound healing assay in MCF-7 and MDMB231.** The wild type peptide inhibited cell migration while mutants had no effect.


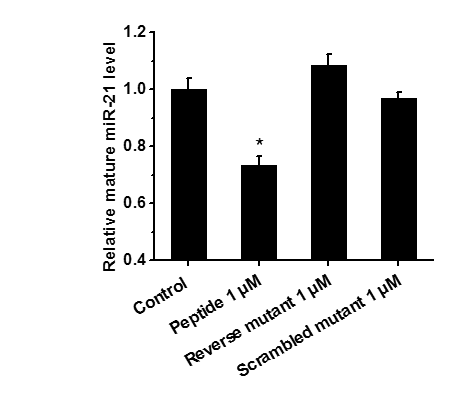


**Figure S13.** Relative levels of mature miR-21 in presence of wild type peptide and mutants (1 µM conc.) in MDMB231 cell line. Error bars represent ± S.D., calculated from three independent experiments.*, p < 0.01 (Student’s *t* test).


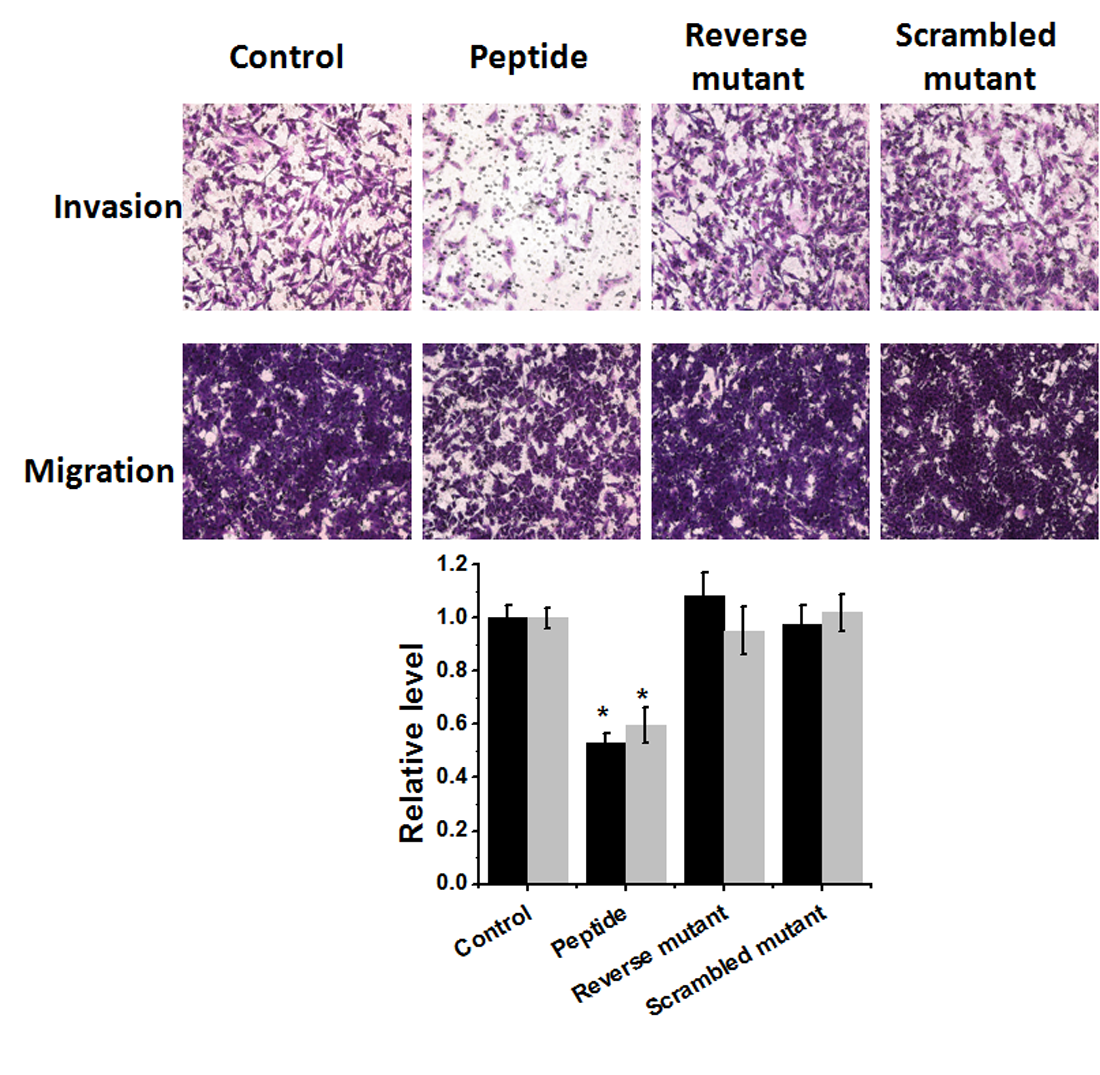


**Figure S14.** **Impact of peptide on cell invasion and migration in MDMB231 cell line**.(A)Peptide treated cells revealed reduced cell invasion and migration, while mutants were ineffective (B) Quantitative analysis showed ~50% reduction in cell invasion(black bar) and ~40% reduction cell migration (grey bar) upon peptide treatment. Error bars represent ± S.D., calculated from three independent experiments.*, p < 0.01 (Student’s *t* test).


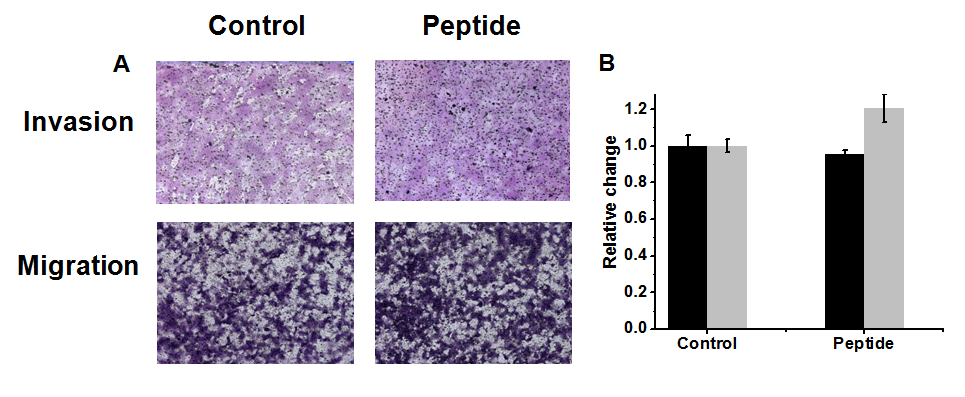


**Figure S15*.* Impact of peptide on cell invasion and migration in HEK293T** (A) Peptide treated cells revealed no change in cell invasion and migration (B) Quantitative analysis. Error bars represent ± S.D., calculated from three independent experiments.
